# The endocannabinoid system and breathing

**DOI:** 10.3389/fnins.2023.1126004

**Published:** 2023-04-18

**Authors:** Beth M. Wiese, Angelica Alvarez Reyes, Todd W. Vanderah, Tally M. Largent-Milnes

**Affiliations:** ^1^Department of Pharmacology, University of Arizona, Tucson, AZ, United States; ^2^College of Medicine, University of Arizona, Tucson, AZ, United States

**Keywords:** endocannabinoid system, respiratory system, opioids, cannabinoids, cannabinoid receptors

## Abstract

Recent changes in cannabis accessibility have provided adjunct therapies for patients across numerous disease states and highlights the urgency in understanding how cannabinoids and the endocannabinoid (EC) system interact with other physiological structures. The EC system plays a critical and modulatory role in respiratory homeostasis and pulmonary functionality. Respiratory control begins in the brainstem without peripheral input, and coordinates the preBötzinger complex, a component of the ventral respiratory group that interacts with the dorsal respiratory group to synchronize burstlet activity and drive inspiration. An additional rhythm generator: the retrotrapezoid nucleus/parafacial respiratory group drives active expiration during conditions of exercise or high CO_2_. Combined with the feedback information from the periphery: through chemo- and baroreceptors including the carotid bodies, the cranial nerves, stretch of the diaphragm and intercostal muscles, lung tissue, and immune cells, and the cranial nerves, our respiratory system can fine tune motor outputs that ensure we have the oxygen necessary to survive and can expel the CO_2_ waste we produce, and every aspect of this process can be influenced by the EC system. The expansion in cannabis access and potential therapeutic benefits, it is essential that investigations continue to uncover the underpinnings and mechanistic workings of the EC system. It is imperative to understand the impact cannabis, and exogenous cannabinoids have on these physiological systems, and how some of these compounds can mitigate respiratory depression when combined with opioids or other medicinal therapies. This review highlights the respiratory system from the perspective of central versus peripheral respiratory functionality and how these behaviors can be influenced by the EC system. This review will summarize the literature available on organic and synthetic cannabinoids in breathing and how that has shaped our understanding of the role of the EC system in respiratory homeostasis. Finally, we look at some potential future therapeutic applications the EC system has to offer for the treatment of respiratory diseases and a possible role in expanding the safety profile of opioid therapies while preventing future opioid overdose fatalities that result from respiratory arrest or persistent apnea.

## 1. Introduction

A functioning respiratory system is critical to survival (Prabhakar and Semenza, 2015; Agrawal and Mabalirajan, 2016) and preserved across many species. The role of the endocannabinoid (EC) system in respiratory homeostasis remains to be fully elucidated. The infancy of our understanding of the interactions of the EC system and respiratory physiology should not equal an assumed lack of influence over each other. Recent studies have shown that administration of a cannabinoid2 receptor (CB_2_R) inverse agonist (Wiese et al., 2020) or a brain penetrant cannabinoid1 receptor agonist (CB_1_R; Wiese et al., 2021) induced respiratory depression (Wiese et al., 2021) – suggesting a tonic role of the CB_2_R and CB_1_R in modulation of respiratory functionality. The EC system has repeatedly been shown to play a holistic regulatory role in many other activities, from experiencing pleasure, to cognitive abilities, and even in the perception of pain (Gardner and Vorel, 1998), making it no surprise that the EC system is also involved in respiratory behavior. It is well established that cannabinoids from the cannabis plant act on our EC system, lending to the discovery of the EC system itself (Gaoni and Mechoulam, 1964). To date all but three states in the US participate in some form of legal cannabis access (Control CfD, 2017), and a recent Gallup poll showed that ~12% of US adults report consistently smoking cannabis (Hrynowski, 2019). Researchers have long been working to understand the impact of cannabis on the body as well as in combination with other medication therapies, including opioids (Epstein and Preston, 2015; Hurd et al., 2015; Jicha et al., 2015; Manini et al., 2015; Mayet et al., 2015; Hrynowski, 2019). While some observational studies have found conflicting results (Ghasemiesfe et al., 2018; Darke et al., 2019), studies since have been able to delineate some of the changes cannabis smoke can have on the body, such as upper lobe emphysematous changes (Gates et al., 2014), hyperinflation (Hancox et al., 2022), bronchiolitis (Gates et al., 2014), alveolar cell hyperplasia with atypia and fibrosis (Darke et al., 2019), sputum production and increased cough (Ghasemiesfe et al., 2018). It is worth stating that this was seen with traditional combustion delivery methods, as opposed to other routes of cannabis administration, and no evidence to date suggests cannabis smoke leads to chronic obstructive pulmonary disease like tobacco smoke does (Owen et al., 2014). But these changes alone do not paint the full picture of the role the EC system has in respiratory functionality. With the increasing number of people utilizing cannabis and cannabinoids on their own or as an adjunct to other treatments (Mauro et al., 2019; Goodwin et al., 2021), especially in combination with analgesics, it is imperative to understand how the EC system and cannabinoids influence our respiratory system.

**Figure 1:**
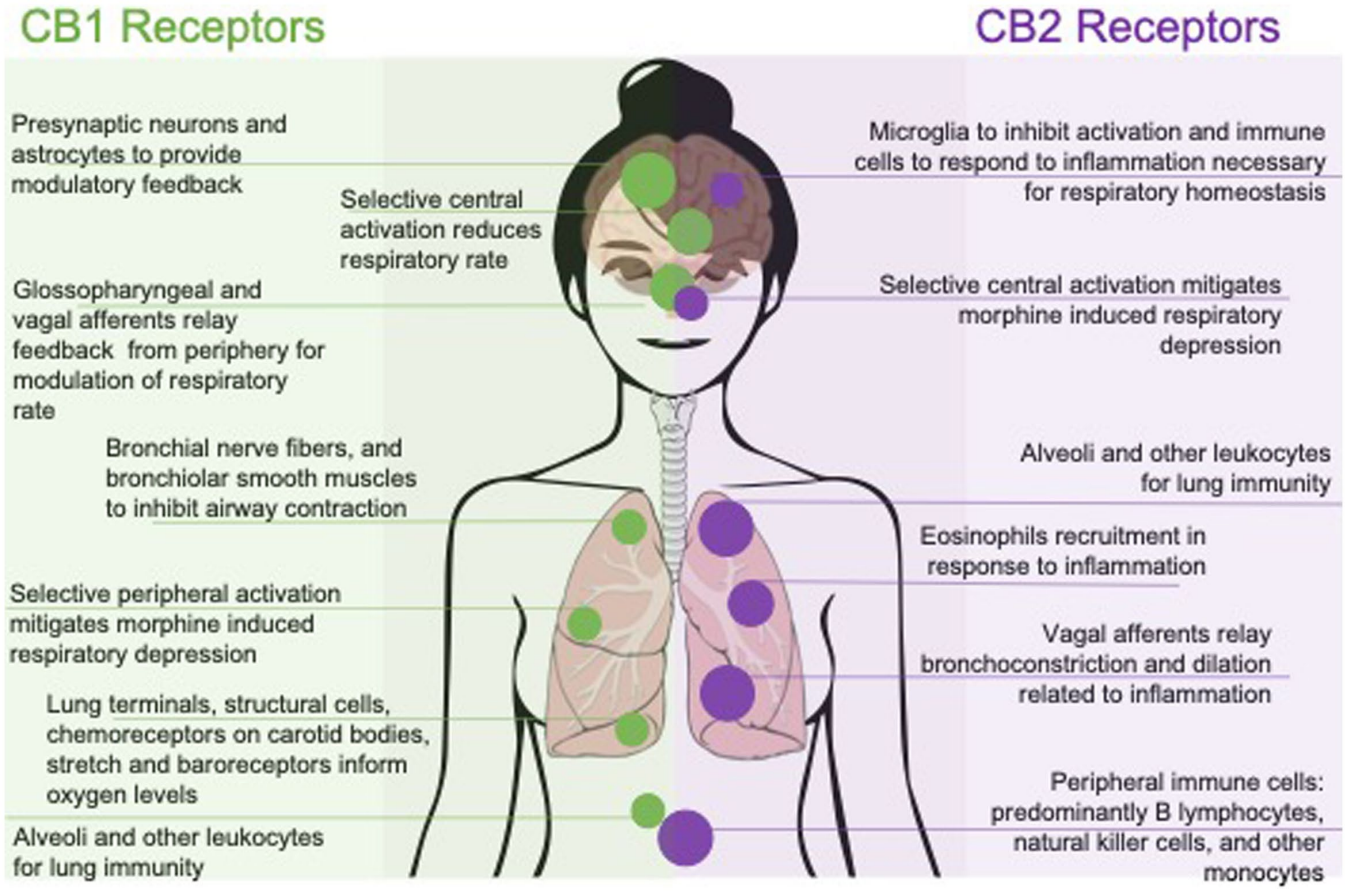
Effects of pharmacologically targeting central or peripheral CB1 and CB2 receptors on respiratory function. Respiratory outcomes are represented by their mechanism of action; with CB1 selective affinity to the left and CB2 selective affinity to the right. Outcomes are also represented with peripherally mediated outcomes along the bottom and centrally, or systemic outcomes, along the top.

This review will explore cannabinoids and the EC system in the context of respiratory regulation, highlighting CB_1_R and CB_2_R influence in the context of central versus peripheral activation, followed by the effects of organic and synthetic cannabinoids on breathing. A summary of cannabinoids effects on breathing is laid out in Figure 1. While additional research is available on cannabinoid tolerance (Deng et al., 2015; Bradford and Bradford, 2016; Bradford et al., 2018; Wiese and Wilson-Poe, 2018; Soliman et al., 2021) or sex differences (De Fonseca et al., 1994; Craft et al., 2013; Channappanavar et al., 2017; Cooper and Craft, 2018; Gargaglioni et al., 2019; Silver and Hur, 2020; Albrechet-Souza et al., 2021; Boullon et al., 2021; Levine et al., 2021; Silveyra et al., 2021; Llorente-Berzal et al., 2022; Simone et al., 2022) could affect breathing, they are outside the scope of this review. We will discuss future possible therapeutic applications for treatment of respiratory diseases as well as a possible role in preventing future opioid overdose fatalities that result from respiratory arrest or persistent apnea.

The respiratory system is made up of two main components, a reflexive and cognitive component. The reflexive component is always at work; meticulously monitoring carbon dioxide (CO_2_) levels, pH changes, and expelling waste 24/7 without any conscious thought or input (Guyenet, 2011; Ogoh, 2019). The other component is the cognitive side. The reflexive component can be overridden and altered by a conscious choice to intervene such as when engaging in breathing exercises, holding ones’ breath, or smoking of a substance (Evans et al., 1999; Mckay et al., 2003; Butler, 2007; Raux et al., 2007). It is these intentional altered inhales that aid the gas exchange of inhaled compounds from the lungs into the bloodstream, such as cannabis or other inhalants The effects felt through inhalation are almost immediate thanks to the efficiency of this fine-tuned respiratory system (Hickey, 2020). The most common route of cannabis and synthetic cannabinoid (SC) consumption is through inhalation, making it vital to public safety that we understand the effects these compounds have on lung tissue and function, as well as how our endogenous cannabinoids influence our respiratory behavior for future therapeutic discovery.

## 2. Central and peripheral respiratory influence of cannabinoids and the endocannabinoid system

### 2.1. Cannabinoids and the endocannabinoid system

**Figure 2:**
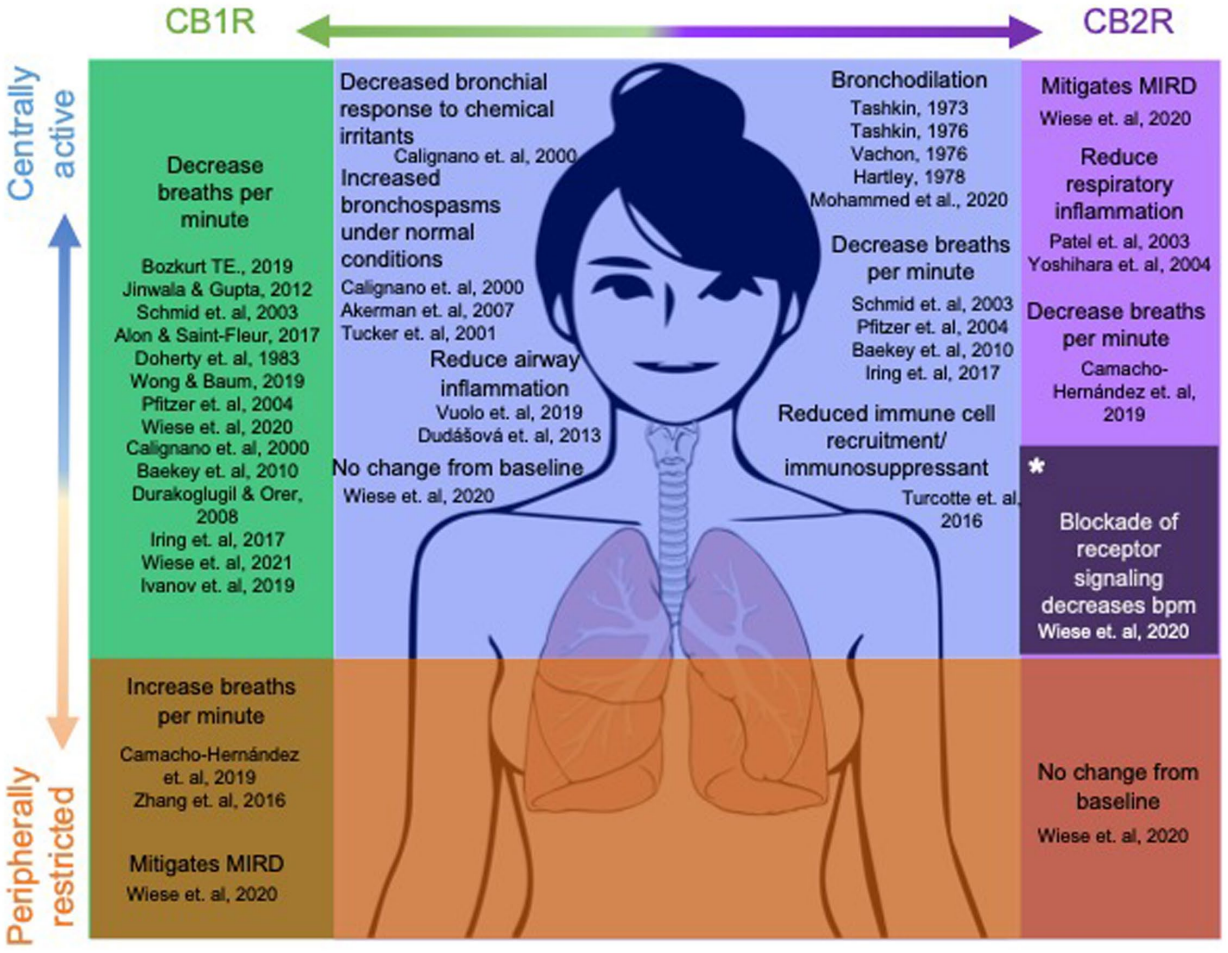
CB1/CB2 receptor distribution and current understanding of their role in respiratory function. Dots in the brain represent centrally mediated effects, dots in the lungs and abdomen represent peripherally mediated effects. Dot size corresponds to concentration levels of the receptor within the region.

The EC system is highly integrated in multiple organ systems of the brain and body, and involved in multiple ways in all homeostatic regulation (Evans et al., 1999; Mckay et al., 2003; Butler, 2007; Raux et al., 2007; Guyenet, 2011; Soliman et al., 2021). Both cannabinoid receptors, CB_1_R and CB_2_R, have varying distributions in the body, purporting different roles between the two. Within the central nervous system (CNS) CB_1_R are primarily localized within the CNS on presynaptic cells for inhibitory feedback to the cell (Ghasemiesfe et al., 2018), as well as some non-neural tissue. CB_2_Rs are involved in inflammation and immunology in the periphery, as well as the CNS, where they are highly expressed in immune cells (Galiègue et al., 1995) and microglia (Schmöle et al., 2015) on the CNS, regulating immune functions (Soliman et al., 2021). With the ubiquitous distribution of cannabinoid receptors within the CNS, especially the CB_1_R, it is no surprise that the EC system plays a direct role in fine tuning the process of breathing and can be manipulated by exogenous or endogenous cannabinoids. Locations of known EC system influence are shown in Figure 2. While drug administration through inhalation is fast and effective, cannabinoids have been shown to influence respiratory rate through other routes of administration (Ryberg et al., 2007; Webster and Schmidt, 2019), as well as offering a more personalized treatment plan for patients who do not tolerate inhalation, or an alternative to traditional combustion based methods of drug delivery. While reports of direct CNS administration of the dual CB1/CB_2_R agonist, WIN 55212-2, produced respiratory depression (Pfitzer et al., 2004), imagining studies for cannabinoid receptors have been inconsistent in confirming receptor presence and exact concentration levels in brainstem respiratory nuclei (Ryberg et al., 2007; Huxtable et al., 2010; Lorea-Hernandez et al., 2016).

The most studied and well understood EC lipids are anandamide (AEA), a partial agonist, and 2-arachidonoylglycerol (2-AG), a full agonist (Soliman et al., 2021), at both CB_1_R and CB_2_R. Both ligands are produced on demand in the postsynaptic cell for retrograde regulation of presynaptic activity and glial cell function. Endogenous cannabinoid ligands, AEA and 2-AG, are synthesized intracellularly (Matias et al., 2002), further supporting a critical role of the EC system in the homeostatic process. Cannabinoids and EC lipids are not the only active compounds to consider about this system, as some of their metabolites, like 2-arachidonoyglycerol ether (Levine et al., 2021), have also been shown to activate and modulate CB_1_R and CB_2_R (Turcotte et al., 2016). Other endogenous molecules have been shown to activate CB_1_R and CB_2_R, such as oleamide, N-oleoyl dopamine, N-arachidonoyl-dopamine, O-arachidonoyl-ethanolamine, noladin, and virodhamine, just to name a few (Porter et al., 2002; Ralevic, 2003; Bradshaw and Walker, 2005); for review of EC metabolites see (Pertwee, 1997; Bradshaw and Walker, 2005; Turcotte et al., 2016). All of these lipids predominantly target the CB_1_R and CB_2_R, but have been shown to bind to other targets such as orphan receptors; GPCR GPR-55 (Ryberg et al., 2007; Turcotte et al., 2016), GPR18 (McHugh et al., 2012), GPR110 (Lee et al., 2016), GPR119 (Brown, 2007) transient receptor potential channels (TRP; De Petrocellis et al., 2012), and peroxisome proliferator-activated receptors (PPARs; Brown, 2007; Bozkurt, 2019). Orphan GPCRs which respond to cannabinoid ligands have emerged as putative cannabinoid receptors. While cannabinoids have been shown to bind to these GPCRs and ion channels, their effect on breathing have yet to be fully elucidated.

Both CB_1_R and CB_2_R are GPCRs that negatively couple to adenylyl cyclase, activate potassium (K^+^) channels, and inhibit calcium influx to hyperpolarize the cell and attenuate vesicular release (Schmid et al., 2003; Yoshihara et al., 2004; De Petrocellis et al., 2012; Jinwala and Gupta, 2012; Gamage et al., 2018; Bozkurt, 2019). CB_1_R and CB_2_R also stimulate the mitogen-activated protein kinase (MAPK) pathway (Carroll et al., 2012; Jinwala and Gupta, 2012), potentially explaining how this system communicates to recruit necessary support cells to regulate neuronal behavior. Specifically, agonism of CB_1_Rs activates the MAPK pathway, impacting cell transcription, translation, motility, shape, proliferation, and differentiation from the resulting phosphorylation of nuclear transcription factors, and that can cause CB_1_R desensitization and internalization if prolonged (Carroll et al., 2012; Jinwala and Gupta, 2012). For a list of receptors and location see table 1 by Bozkurt (2019) review (Bozkurt, 2019). Given the wide-spread distribution of CB_1_R and CB_2_Rs, understanding their contributions to respiratory control can be evaluated by peripheral versus central contributions.

CB_1_R activation in the periphery is involved in the functional reactivity of the airways through stimulation that inhibits the contraction of airway smooth muscle *via* acetylcholine inhibition from cholinergic nerves (Bozkurt, 2019). The CB_1_Rs are found to couple to Gα_i_ that can lead to GIRK coupling similar to mu opioid receptors (MOR), hyperpolarizing the neurons (Pertwee et al., 2010; Merighi et al., 2012) to reduce respiratory rate (Doherty et al., 1983; Schmid et al., 2003; Alon and Saint-Fleur, 2017) yet, unlike that of opioids, lack the ability to cause a persistent apnea. This is thought to be due to MORs found both pre- and post synaptically (Alon and Saint-Fleur, 2017; Cohen and Weinstein, 2018) producing additional inhibitory feedback while CB_1_Rs are predominantly found presynaptically (Vanderah, 2007; Tree et al., 2010). Additionally, the ability to track and observe our oxygen (O_2_) saturation and respiratory rate appear to be a CB_1_R driven effect, yet whether the CB_1_R effect is peripherally or centrally mediated remains to be uncovered.

Literature has also shown CB_2_Rs enhance the release of anti-inflammatory factors, as well as modulate respiratory drive (Komorowska-Müller and Schmöle, 2020). Complimentary qRT-PCR assay confirmed heavy populations of CB_2_Rs in the preBötzinger complex (pBc; Schmöle et al., 2015; Wiese et al., 2020); unpublished immunohistochemistry assays revealed an abundance of CB_2_Rs co-localized with Iba1, not GFAP, suggesting their location to be on microglia and not astrocytes (unpublished data, Largent-Milnes lab) aligning with prior reports. Activated microglia decrease the amplitude of nearby neuron action potentials, including those of the pBc (Camacho-Hernández et al., 2019) suggesting a possible protective mechanism for CB_2_R agonists to inhibit activated microglia. CB_2_R have also been found on multiple types of immune cells, including white blood cells, B lymphocytes, natural killer cells (Fernández-Ruiz et al., 2007), polymorphonuclear leukocytes such as eosinophils (Oka et al., 2004; Chouinard et al., 2011, 2013) and other monocytes (Galiègue et al., 1995). They are implicated in inflammatory responses of the periphery and CNS, acting through sensory nerves (Ogoh, 2019). The predominant cell type expressing CB_2_R are B lymphocytes (Fernández-Ruiz et al., 2007), with the level of expression contingent on the type and strength of the stimuli (Muller et al., 2018). Robust localization of CB_2_R in immune cells may purport a role for immunosuppression. Studies across several disease states have shown the role of cannabinoids in immunosuppression through induction of apoptosis, inhibition of cell proliferation, inhibition of the production of cyto- and chemokines, reduce cytokine activation and T cell proliferation, as well as induction of regulatory T lymphocytes. Antagonists of the CB_2_R have shown to prevent THC-induced apoptosis (Fernández-Ruiz et al., 2007), while antagonism of the CB_1_R failed to show similar results (Malfait et al., 2000; Croxford and Miller, 2003) further highlighting the immunoprotective effects of the CB_2_R (Fernández-Ruiz et al., 2007). Chronic inflammatory respiratory conditions, such as allergic asthma, recruit eosinophils to the airways in response (Bozkurt, 2019). These densely packed white blood cells with CB_2_Rs that respond to such inflammatory conditions further suggest a homeostatic role of the EC system in respiratory function and warrants continued investigations of ways to manipulate this mechanism therapeutically for people with inflammatory respiratory conditions.

### 2.2. Central respiratory control and the endocannabinoid system

Inspiratory drive comes from the central pattern generator (CPG) in the brainstem *via* the preBötzinger Complex (pBc), a nucleus located in the ventral lateral medulla and part of the ventral respiratory group. The pBc interacts with the dorsal respiratory group and the central termini of the hypoglossal and vagal nerves to generate respiratory rhythm (Del Negro et al., 2018; Varga et al., 2020) In the dorsal region of the pons, also known as the pneumotaxic center, the parabrachial nucleus, containing Kölliker-Fuse nucleus, provides tonic excitatory inputs to the pBc to provide smooth transitions from inspiration to expiration by inhibiting the rhythmic burstlet conversion to motor output bursts arising from the pBc (Varga et al., 2019). The dorsal respiratory group receives inputs from the apneustic center in the lower pons, as well as feedback from the periphery to inhibit expiration and allow for inspiration to, again, occur. The stretch mechanoreceptors from the lungs, diaphragm, and intercostal muscles (Del Negro et al., 2018; Webster and Schmidt, 2019; Varga et al., 2020), as well as inputs from chemoreceptors and baroreceptors of the carotid bodies and aortic arch, all relay this feedback to the nucleus tractus solitarius, heavily populated with CB_1_Rs (Glass et al., 1997), and the dorsal respiratory group, for modulation of respiratory rate. Additional feedback is provided by the vagal and glossopharyngeal nerves to the nucleus tractus solitarius about O_2_, CO_2_, and pH levels from lung mechanoreceptors and peripheral chemoreceptors to further refine the necessary burstlets to maintain O_2_ levels for cell survival.

The pneumotaxic center inhibits the pBc and apneustic center, while the apneustic center promotes activity of the pBc. The pBc then sends signals to inhibit the pneumotaxic center, moving the tongue out of the way *via* the hypoglossal nerve during inspiration (Ghali, 2019). The nucleus ambiguous controls the pharynx, larynx, and soft palate during inspiration, while the nucleus retroambiguus sends signals to the diaphragm and intercostal muscles in response to inspiration and expiration. While cannabinoid receptors were not traditionally thought to exist in respiratory nuclei, recent studies have confirmed their presence in the pBc (Wiese et al., 2020), as well as neighboring regions controlling motor output has been well established (Glass et al., 1997).

The other CPG, is known as the retrotrapezoid nucleus (Eljaschewitsch et al., 2006)/parafacial (Pfitzer et al., 2004) respiratory group, which controls active expiration, during conditions of exercise or high CO_2_ concentrations (Janczewski and Feldman, 2006). The retrotrapezoid nucleus is believed to promote breathing immediately following birth (Shi et al., 2021) and is opioid insensitive since the endogenously released opioids to comfort the mother and baby during the birthing process (Chernick and Craig, 1982) would be detrimental on the opioid sensitive pBc (Gray et al., 1999), making this potentially opioid insensitive region an area of promise for future research into prevention of the deadly effects of over ingestion of opioids. Genetic knockout mice for selective genes that play a role in active expiration as early as birth (Chatonnet et al., 2007), are unable to survive 24 h post-delivery without administration of naloxone to maintain rhythmic properties of the opioid sensitive pBc (Jacquin et al., 1996).

#### 2.2.1. PreBötzinger complex and the endocannabinoid system

The pBc is responsible for the synchronization of the neuronal burstlets that control automatic inspiration, but not expiration. The pBc neurons are characterized neurokinin 1 receptor (NK1) containing cells that are the targets of neurotransmitters such as substance P, GABA and glutamate. These input neurons as well as the pBc neurons themselves are provided support by astrocytes and microglia (Feldman and Del Negro, 2006; Montandon et al., 2011; Anderson et al., 2016; Sun et al., 2019). Substance P activation of NK1 neurons in somatostatin-containing neurons of the pBc is reported to drive bursts, while Mu opioid receptors (MOR) on these same neurons, when activated, inhibit these same events. In addition, the pBc burslet activity for synchronized inspiration has been shown to be modulated by multiple additional receptors including CB_1_Rs and CB_2_Rs (Feldman and Del Negro, 2006; Montandon et al., 2011; Anderson et al., 2016), purinergic receptors, TRP subtype channel 3, α-amino-3-hydroxy-5-methyl-4-isoxazolepropionic acid (AMPA) receptors (Jacquin et al., 1996), *N*-methyl-D-aspartate (NMDA) receptors, developing brain homeobox protein 1 (DBX1) receptors, gastrin releasing peptide receptors, adenosine receptors, nicotinic acetylcholine receptors, and muscarinic acetylcholine receptors (Smith et al., 1991; Feldman and Del Negro, 2006; Montandon et al., 2011; Anderson et al., 2016; Camacho-Hernández et al., 2019).

The pBc and other respiratory nuclei, including the Bötzinger complex just anterior to the pBc, and a subset of neurons in the ventrolateral medulla are heavily populated with MORs (Del Negro et al., 2018), and NK1Rs, which are colocalized on somatostatin neurons within the pBc (Del Negro et al., 2018). A fatal opioid overdose occurs following activation of MORs within the pBc causing desynchronization of burstlets, and consequently respiratory arrest or persistent apnea. This increase in time it takes to synchronize burstlets slows inspiration but has no impact on the frequency of expiration (Feldman and Del Negro, 2006; Sun et al., 2019), eventually resulting in death; this outcome from hyperpolarization following MOR activation can be reversed with the opioid antagonist, naloxone (Montandon et al., 2011). The fact that levels of CB_1_Rs and CB_2_Rs in the central respiratory nuclei are less than MORs is partly due to their restricted expression to only presynaptic inhibition instead of pre- and postsynaptic inhibition like MORs, possibly explaining why no deaths have been reported by cannabis despite the many similarities in actions opioid and cannabinoid receptors share (Glass et al., 1997).

#### 2.2.2. Support cells, the preBötzinger complex, and the endocannabinoid system

Glia, support cells found in the extracellular environment that are involved in neuronal homeostasis, include microglia and astrocytes (Jäkel and Dimou, 2017). Astrocytes have been shown to support this environment *via* Kir4.1 channels that regulate baseline potassium (K^+^) levels in the pBc (Neusch et al., 2006). Kir4.1 is an inwardly rectifying K^+^ channel exclusively expressed in glial cells within the CNS that modulates extracellular K^+^ homeostasis, maintains astrocyte resting membrane potential, and facilitates glutamate uptake (Funk et al., 2015). Additionally, astrocytes have been well defined in supportive roles maintaining water and ion concentrations, blood–brain barrier integrity, and membership in the tripartite synapse (Jäkel and Dimou, 2017). Astrocytes within the pBc are morphologically different compared to astrocytes within the brainstem by their different K^+^ channel expression patterns (Glass et al., 1997; Funk et al., 2015), likely reflective of their role in respiratory modulation (SheikhBahaei et al., 2018). While not fully understood, studies to date suggest astrocytes assist in the exchange of K^+^ and Cl^−^ on neighboring GABAergic neurons and regulate extrasynaptic glutamate concentrations *via* an exchange with cystine. It has also been shown that declines in this glutamate/cystine exchange can promote trafficking of mGLU5 receptors to the extrasynaptic membrane and are believed to be directly involved in the onset of long-term depression. This integrated involvement of astrocytes and the behavior of glutamatergic and GABAergic neurons support evidence that they may be actively involved in respiratory rhythm generation within the pBc. A certain subset of astrocytes within the pBc have demonstrated increased rises of calcium immediately preceding inspiratory neuronal firing (Okada et al., 2012). Inhibition of astrocytes has been shown to depress breathing *in vivo* (Young et al., 2005). Astrocytes in the pBc release ATP which under hypoxic conditions increases respiratory activity (Rajani et al., 2018). Though the exact role of astrocytes in the pBc is poorly understood, it is thought that they modulate the respiratory network (Funk et al., 2015).

Prior studies have shown that either the inhibition (or depletion) of microglia, or their activation, reduces the respiratory rates (Lorea-Hernandez et al., 2016). Beyond extracellular homeostatic maintenance, phenotypically classified as M0, microglia are also critical mediators of neuroinflammation (Hülsmann et al., 2000; Erlichman and Leiter, 2010; Huxtable et al., 2010) and respond quickly to small extracellular K+ changes to become activated (Varga et al., 2020). Once activated, phenotypically classified as M1, microglia mediated neuroinflammation by phagocytizing pathogens, recruiting inflammatory cells, and the production of chemokines and cytokines (Hickman et al., 2018), as well as upregulating adaptive immune responses (Orihuela et al., 2016). Once the threat has dampened, the healing process begins *via* polarization into an M2 microglia phenotype, or alternative activation state. Here microglia begin to express growth factors and anti-inflammatory mediators to aid in recovery (Franco et al., 1988). While three distinct phenotype classification states have been established, it is understood that these states exist on a spectrum and are not an all or none classification. Microglia, when activated, decrease the amplitude of the action potentials in nearby neurons (Camacho-Hernández et al., 2019) so microglia activation in the pBc could have a negative impact on burstlets that reach burst threshold.

Further research has uncovered CB_2_R knockout (KO) mice to be unable to fully polarize to an M2 microglia phenotype (Komorowska-Müller and Schmöle, 2020), further supporting the necessity of CB_2_Rs to facilitate polarization to an M2 phenotype. The M2 phenotype is further stratified into M2a and M2c activation states. Stimuli typical of these activation states have been shown to increase synthesis of endogenous EC ligands such as 2-AG and AEA further suggesting a role for the EC signaling system (Mecha et al., 2015) in the M2 phenotype and anti-inflammatory effects. Studies comparing administration of AEA as well as administration of a CB1/2 receptor agonist, such as WIN 55212-2, has also shown to suppress proinflammatory cytokines such as IL-5 and inducible nitric oxide synthetase. Conversely, inhibition of CB_2_R in the setting of inflammation is known to exacerbate neuronal damage more so than CB_1_R inhibition allowing microglia to pursue a pro-inflammatory response (Eljaschewitsch et al., 2006). Despite initial reports that CB_2_R expression was present only in the periphery, studies have shown CB_2_R expression in the brain in both pathological and nonpathological conditions (Schmöle et al., 2015). Furthermore, the implication of ECs producing an anti-inflammatory state postulates a role for ECs in respiratory homeostasis within the CNS (Cabral et al., 2008; Montandon et al., 2016; Varga et al., 2020). For a review of microglia CB_2_Rs see (Komorowska-Müller and Schmöle, 2020).

### 2.3. Peripheral respiratory control and the endocannabinoid system

In the periphery, receptors and neurotransmitters work together to regulate sympathetic activation instead of the maintenance mechanisms seen in the CNS. Among the cannabinoid receptors, CB_1_R plays a role in the functional reactivity of the airways through stimulation that inhibits the contraction of airway smooth muscle *via* inhibition of acetylcholine from cholinergic nerves (Bozkurt, 2019). It is believed that AEA activation of peripheral CB_1_Rs is one means to control bronchial contractility. This control is dependent on the current state of the bronchial muscle. During the capsaicin-evoked bronchospasm, when the muscle is contracted, AEA can ease this contraction, likely by inhibiting prejunctional release of excitatory neurotransmitters and neuropeptides (Bozkurt, 2019). Alternatively, bronchoconstriction can be seen because of CB_1_R activation when the smooth muscle is relaxed following the removal of a constricting influence on the vagus nerve (Calignano et al., 2000). CB_2_Rs are likely to play a role in the mechanisms for neurogenic inflammation, acting through sensory nerves (Bozkurt, 2019). Chemoreceptors located on carotid arteries respond to changes in blood O_2_ levels, baroreceptors sense blood pressure changes, and activated pulmonary stretch receptors release surfactant, reducing surface tension for the transition to expiration (Calignano et al., 2000). These stretch receptors interact with chemoreceptors and baroreceptors to continuously inform the central respiratory centers, *via* the vagal and glossopharyngeal nerves, to maintain respiratory homeostasis (Rice et al., 1997; Calignano et al., 2000; Niederhoffer et al., 2003; Schmid et al., 2003). ECs are produced and their receptors are expressed in each of these areas.

As a first line of defense, the respiratory system contains numerous immune cells, the abundance of those cells being alveolar macrophages (Adams et al., 2017) to protect us against aerosolized bacteria, viruses and toxins are bronchial epithelial cells, alveolar macrophages and dendritic cells of the lungs (Turcotte et al., 2016). While most abundant in alveoli, other leukocytes express CB_1_Rs and CB_2_Rs and play a role in lung immunity (Turcotte et al., 2016). Recent literature has begun to uncover the role the EC system plays in this line of defense. CB_1_Rs and CB_2_Rs have been found *via* mRNA and proteins (Galiègue et al., 1995) detection in vagal afferents (Niederhoffer et al., 2003), nerve fibers that innervate bronchioles (Calignano et al., 2000), and bronchiolar smooth muscle cells (Pertwee, 1997; Ständer et al., 2005; Izzo and Sharkey, 2010; Szymaszkiewicz et al., 2018), as well as the peripheral termini of lung tissue (Rice et al., 1997; Calignano et al., 2000; Niederhoffer et al., 2003; Schmid et al., 2003) and are believed to play a homeostatic role in bronchial contractility (Calignano et al., 2000). This is important given that current literature has not reported CB_1_Rs and CB_2_Rs expression in the epithelial cells of the primary airway despite the presence of their mRNA being found in the human bronchial epithelial cell line, 16HBE14o (Turcotte et al., 2016). Recently the lung tissue of patients with adenocarcinoma was utilized to isolate macrophages and revealed CB_1_Rs and CB_2_R mRNA and proteins in macrophages associated with the tumor and non-tumor collected samples (Staiano et al., 2016). Levels of CB_2_R were higher than CB_1_R in alveolar and monocyte macrophages, but they were found to be functionally opposite in extracellular signal-regulated kinases ½ (ERK1/2) phosphorylation assays. Airway epithelial cells are part of that first line of defense that can identify pathogens and activate leukocytes in conditions of inflammation (Weitnauer et al., 2016) making their role and response to cannabinoids and ECs of great interest for future directions and potential future therapies. Thus, highlighting the multitude of outcomes the EC system can produce through different mechanisms within the respiratory defense system of the periphery.

The ability of the mammals to track and observe our O_2_ saturation and respiratory rate appear to also be a CB_1_R driven effect, but the mechanisms underlying these effects remain unknown. In one study the cannabinoid reuptake inhibitor, AM404, was administered to investigate the effects of the endogenous cannabinoids on breathing. This increase in cannabinoid availability reduced respiratory rate and arterial O_2_ saturation. This effect was completely abolished in CB_1_R KO mice (Iring et al., 2017). While other studies have reported respiratory benefits from peripherally restricted CB_1_R agonism when coadministered alongside morphine (Wiese et al., 2021). These data lend support for further investigation of peripheral versus brain penetrant CB_1_R agonism to play different roles in respiratory functionality. Below we detail some of these actions in the periphery.

#### 2.3.1. Chemoreceptors and baroreceptors and the endocannabinoid system

Cannabinoids may act on peripheral sites such as on chemoreceptors and baroreceptors. Through *in-situ* hybridization, CB_1_Rs have been found to have some expression in the nodose-petrosal-jugular ganglia, superior cervical ganglia, and some sparse localization within the carotid body (Roy et al., 2012). Chemoreceptors are what sense gas and pH levels. They respond when O_2_ levels rise and fall, as well as CO_2_ levels. They also provide feedback on pH levels in the blood so alterations can be made if necessary. Central chemoreceptors are located below the ventrolateral surface of the medulla. As arterial partial pressure of carbon dioxide (P_CO2_) rises, it diffuses across the blood brain barrier to raise the CO_2_ content of cerebrospinal fluid where it eventually hydrates to carbonic acid and ionizes to reduce the pH of cerebrospinal fluid. These pH sensitive receptors within the medulla detect this change and release L-glutamate (Mifflin, 1992), along with ATP, relay to the pBc to increase the respiratory rate in order to decrease arterial P_CO2_ (Machado and Bonagamba, 2005; Braga et al., 2007). It has also been suggested that respiratory depression resulting from CB_1_R activation may involve peripheral arterial chemoreceptors; other studies have reported a protective benefit from peripheral CB_1_R activation (Wiese et al., 2021) leaving future studies to fully parse out the role of the peripheral CB_1_R. Expression of CB_1_R within the carotid body implicates a role for blood flow regulation thereby affecting respiratory control. Central chemoreceptors do not directly respond to partial pressure of oxygen (P_O2_), only P_CO2_. Peripheral chemoreceptors, on the other hand, which are located on carotid and aortic bodies, are stimulated by increased P_CO2_, decreased blood pH, and decreased P_O2_ (Machado and Bonagamba, 2005) to alert the central respiratory centers of necessary alterations needed to maintain homeostasis. In addition, CB_1_Rs can act through the carotid body by forming heterodimers with other GPCRs present such as delta opioid receptors, MOR, adenosine 2A receptors, and dopamine 2 receptors (Porzionato et al., 2018).

Baroreceptors are rapid acting mechanoreceptors located in the carotid sinus and aortic arch sensing changes in arterial blood pressure. Previous studies imply that increases in blood pressure may abruptly prolong expiration in response to baroreceptor activation (Baekey et al., 2010). Cannabinoids may also act *via* a modulating role in the baroreceptor reflex. Activation of cannabinoid receptors in the nucleus of tractus solitarius (Distribution Po, n.d.; Cohen and Weinstein, 2018), through administration of WIN 55212-2 and CP 55940, elicits a baroreflex-like response through a decrease in arterial pressure and sympathetic inhibition, which is antagonized with pretreatment of the CB_1_R antagonist, AM281 (Baekey et al., 2010). An intact baroreceptor reflex was required to demonstrate the baroreflex-like response as sino-aortic-denervated rats demonstrated attenuated responses to WIN 55212-2, implicating a more modulatory role of cannabinoids (Durakoglugil and Orer, 2008).

Few studies have focused solely on synthetic cannabinoids (SCs) effects on peripheral receptors within the respiratory system, such as chemoreceptors and baroreceptors. Prior literature has shown activation of chemo- and baroreceptors can increase bronchial airway resistance, reducing overall respiratory functions. This has been explored as a possible mechanism of central CB_1_R stimulation to explain the respiratory depression seen from SCs (Alon and Saint-Fleur, 2017).

#### 2.3.2. Lung tissue and the endocannabinoid system

Cannabinoids, both exogenous and endogenous, have shown to have potentially therapeutic benefits due to their inhibitory effects on immune functions and cell recruitment in lung inflammation. Conversely, cannabinoids have also shown to slow respiratory pathogen clearance and be deleterious on lung function (Turcotte et al., 2016). Other conflicting findings have shown an absence of cannabinoid effects altogether, but many of these studies were conducted in naïve animals, while studies in pathological models have demonstrated beneficial effects from cannabinoids (De Petrocellis et al., 2017).

Lung tissue terminals, structural cells, and leukocytes (Turcotte et al., 2016) have all been shown to contain CB_1_Rs (Rice et al., 1997; Calignano et al., 2000) and control bronchial contractility and function in a homeostatic role (Calignano et al., 2000). In one study, capsaicin-evoked bronchospasm was relaxed following a local administration of a CB_1_R agonist *via* inhibition of vagal input, suggesting CB_1_Rs role as a homeostatic respiratory regulator (Calignano et al., 2000). This promotion of homeostatic respiration by peripheral CB_1_R activation may explain how a peripherally restricted CB_1_R agonist can have a different effect than a brain penetrant CB_1_R agonist (Wiese et al., 2020, 2021). Alveolar macrophages extracted from people who consume cannabis *via* combustion regularly have decreased capability to ingest/remove staphylococcus aureus (Baldwin et al., 1997), produce less nitric oxide (Shay et al., 2003), and caused a weakened host defense through decreased cytokine priming (Roth et al., 2004). Following capture of these antigens, dendritic cells migrate to lymph nodes to pass the antigen to naïve T cells (Turcotte et al., 2016). CB_2_Rs have been shown to facilitate this migration to the lymph nodes (Lu et al., 2006). This migration process becomes impaired following tetrahydrocannabinol (THC) exposure and could leave the individual open to impaired immune responses from pulmonary pathogens (Lu et al., 2006). As with the rest of the EC system, there are still future studies necessary to fully uncover the mechanisms by which cannabis consumption impacts the respiratory immune system as other data have found benefits from THC on the severity of acute respiratory distress syndrome through alterations of lungs microbiota (Mohammed et al., 2020).

#### 2.3.3. Cranial nerves and the endocannabinoid system

The sensory nerves of the respiratory system include the vagal, glossopharyngeal, phrenic, and intercostal nerves (Del Negro et al., 2018). The vagal and glossopharyngeal nerves relay all the necessary information in response to peripheral respiratory actions to the central respiratory nuclei to make needed modifications and signal when to transition to the next phase in the respiratory cycle. Expression of CB_1_Rs within nuclei of the glossopharyngeal and vagal nerve have suggested a peripheral role in sensory and autonomic function for ECs (Burdyga et al., 2004; Yoshihara et al., 2004; Ye et al., 2019). The phrenic and intercostal nerves send information from the diaphragm while the internal intercostal nerves relay additional stretch information from the intercostal muscles. These nerves bring in information from mechanoreceptors that sense pressure and stretch changes in the lungs, as well as O_2_ saturation, CO_2_ saturation, and pH levels *via* chemoreceptors all to fine tune the respiration sequence (Del Negro et al., 2018).

## 3. Organic and synthetic cannabinoids on breathing

The most common, and well known organic cannabinoid is Δ^9^-tetrahydrocannabinol (THC), a mixed CB_1_R/CB_2_R partial agonist (Makriyannis, 2014; Nikas et al., 2015), that does not cause respiratory depression (Tree et al., 2010), and has been shown to be beneficial in the treatment of chronic pain, migraines, anorexia, nausea, just to name a few (Wills and Parker, 2016; Yuill et al., 2017; Wiese and Wilson-Poe, 2018; Mohammed et al., 2020). The experienced psychoactive effects seen with cannabis use are largely attributed to the result of THC activation on the multiple receptor targets it may occupy, including CB_1_R, CB_2_R, as well as GPR55 (Ryberg et al., 2007), GPR18 (McHugh et al., 2012), serotonin 3A (Barann et al., 2002), PPARγ (Vara et al., 2013), and TRP channels 2, 3, and 4 (De Petrocellis et al., 2011, 2012), explaining just how THC can have such a wide spectrum of therapeutic benefits for such a broad list of ailments, as well as impacts on cognitive functioning, motor movements, and possible immunosuppression (Vachon et al., 1976; Calignano et al., 2000; Owen et al., 2014). Medicinal benefits of THC also appear to be easily modulated by other cannabinoids (LaVigne et al., 2021), for review see (Morales et al., 2017), making fine tuning individual therapies with THC a very promising and future public health benefit. Multiple studies have shown in healthy volunteers and volunteers with chronic bronchial asthma, of minimal or moderate severity, that the use of THC results in bronchodilation (Lee et al., 2001; Croxford and Miller, 2003; Vanderah, 2007; Rieder et al., 2010), and the concentrations of THC that demonstrate this protective finding are concentrations that do not result in central or cardiovascular effects (Tashkin et al., 1973, 1976; Vachon et al., 1976; Hartley et al., 1978) suggesting a possible peripherally driven mechanism. Conversely, disruption of the alveolar epithelium and vascular endothelium of any kind is known as an acute lung injury (Lu et al., 2006). Under these conditions the use of cannabinoids as a treatment option proved beneficial in all (Ribeiro et al., 2015; Fujii et al., 2019) but one study showed CBD to be pro-inflammatory under these conditions (Karmaus et al., 2013).

Another common organic cannabinoid is cannabidiol (CBD). CBD has also shown promising effects, likely also due to the promiscuous affinity CBD has to multiple receptors, for review see (Morales et al., 2017). Studies of systemic administration have shown that CBD reduces the inflammation response and structural changes that take place during the remodeling process of asthma (Vuolo et al., 2019), as well as stunt inflammatory parameters following acute lung injury (Ribeiro et al., 2015). Reductions in airway responsiveness have also been observed following CBD treatment (Vuolo et al., 2019). CBD also influences airway smooth muscle tone and reduces contractions caused by endogenous cannabinoids suggesting beneficial effects for the treatment of obstructive airway disorders (Dudášová et al., 2013). Furthermore, in respiratory studies, CBD was shown to prevent morphine-induced respiratory depression in room air but lost those protective effects under a CO_2_ challenge (Wiese et al., 2021).

Synthetic cannabinoids (SCs) are a class of cannabinoids that were developed by chemists to investigate and further understand the EC system (Pertwee, 1997; Carroll et al., 2012; Yuill et al., 2017; Szymaszkiewicz et al., 2018). They were not designed for human consumption (Jinwala and Gupta, 2012), as many of these compounds are selective to the CB_1_R with an ability to cross the blood–brain barrier and can be dangerous (Gunderson et al., 2014), while some experiences have been unpleasant to the person, examples of these compounds being utilized by people outside the laboratory have been reported as case studies (AAPCC, n.d.; Gunderson et al., 2014; Tait et al., 2016; Alon and Saint-Fleur, 2017; Cohen and Weinstein, 2018; Darke et al., 2019; Mathews et al., 2019) and have been equally important in understanding the mechanisms by which the EC system functions (Schmid et al., 2003; Robson, 2005; Jinwala and Gupta, 2012; Trecki et al., 2015; Adams et al., 2017; Tournebize et al., 2017; Ivanov et al., 2019). An overview of these different outcomes on breathing can be seen in Figure 1. Preclinical and clinical studies have shown CB_1_R brain penetrant SCs to result in respiratory depression (Schmid et al., 2003; Jinwala and Gupta, 2012; Alon and Saint-Fleur, 2017; Wong and Baum, 2019). Inhalation of SCs can damage bronchiolar epithelium and the protective surfactant layer within alveoli causing hypoxia and acidosis from the resulting interference in effective gas exchange (Pertwee et al., 2010). These results have been shown to influence respiratory function by increasing airway resistance (Alon and Saint-Fleur, 2017) and reductions in blood pressure and circulating noradrenaline resulting in sympathetic inhibition and increased vagal tone (Niederhoffer et al., 2003; Schmid et al., 2003). SCs have also been shown to suppress cough and bronchospasms through inhibition of the excitatory effects of noradrenaline in the airways, which may provide an explanation for respiratory depression through vagal transmission (Calignano et al., 2000). Additionally, other adverse effects have been seen with the use of SCs such as tachycardia, paranoia, acute kidney injury, seizures, nausea and vomiting, calls to poison control, and trips to the emergency room (Tait et al., 2016; Cohen and Weinstein, 2018; Darke et al., 2019; Mathews et al., 2019). If peripheral CB_1_Rs also assist in the suppression of respirations, this may be the mechanism at which they are able to do so (Calignano et al., 2000). Yet, other studies have found protective benefits from selective CB_1_R activation in combination with morphine (Wiese et al., 2021). Since many phytocannabinoids, as well as mixed cannabinoid agonists, also show an affinity for the CB_1_R but do not induce respiratory depression (Wiese et al., 2020), understanding how CB_1_R activation drives respiratory depression is vital to ensuring safe consumption of these opioid adjuncts.

While SCs have shown respiratory depression through CB_1_R activation in prior studies, there has not been a clear delineation as to whether these effects are directly a cause of central or peripheral CB_1_R activation (Pfitzer et al., 2004). The CB_1_R mechanism of action is similar to the MOR to reduce the neuron’s ability to depolarize (Pertwee et al., 2010; Merighi et al., 2012) and lends itself that selective, central CB_1_R activation could induce respiratory depression (Doherty et al., 1983; Schmid et al., 2003; Alon and Saint-Fleur, 2017). Furthermore, with only presynaptic CB_1_Rs, compared to MORs that are found on pre and postsynaptic terminals (Lopez-Moreno et al., 2010; Scavone et al., 2010), may explain why fatal respiratory depression has not been seen from central CB_1_R activation compared to MOR activation in this region. As with other potent cannabinoid agonists at the CB_1_R (Gunderson et al., 2014; Ivanov et al., 2019), SCs activate the MAPK pathway, impacting cell transcription, translation, motility, shape, proliferation, and differentiation from the resulting phosphorylation of nuclear transcription factors, that if prolonged, can cause CB_1_R desensitization and internalization (Carroll et al., 2012; Jinwala and Gupta, 2012) Administration of the cannabinoid, WIN 55212-2, a mixed CB_1_R/CB_2_R agonist, in preclinical models has been shown to produce a depressed effect on respirations (Schmid et al., 2003; Pfitzer et al., 2004). Following the inhibition of respiratory depression with the administration of SR-141716, a CB_1_R inverse agonist, it was concluded that the depressive effect was a CB_1_R mediated mechanism (Pfitzer et al., 2004).

Prior literature has shown that CB_1_R activation reduces airway contraction and cholinergic induced contractions, while still providing an improvement of static lung elastance and reduced collagen fiber content helping to keep the alveoli from collapsing (Wang et al., 2016). However, other studies have postulated other mechanisms of the pulmonary pathways (Tucker et al., 2001; Akerman et al., 2007). In a condition of capsaicin induced cough, the endogenous cannabinoid, AEA, inhibited the cough response as well as the associated bronchoconstriction, but when administered on its own induced bronchoconstriction (Calignano et al., 2000). These effects were only reversed following the administration of the CB_1_R inverse agonist, SR-141716, suggesting a CB_1_R mediated effect. It is worth noting that AEA has been shown to activate TRPV receptors, giving pause for speculation that these results were completely CB_1_R driven (Tucker et al., 2001; Akerman et al., 2007).

In one study using intraesophageal HCl instillation to assess cannabinoid receptor inhibitory effects on the sensory nerve pathways involved in bronchoconstriction and airway microvascular leakage found administration of WIN 55212-2 (CB_1_/CB_2_ agonist) or JWH 133 (CB_2_R agonist) abolished all associated neurogenic inflammation (Cui et al., 2007). These data support the prior literature that has found administration of the CB_2_R agonist, JWH 133, inhibits citric acid induced coughing (Patel et al., 2003) and main bronchi contraction induced by capsaicin in preclinical models (Yoshihara et al., 2004). These findings all suggest a role for the CB_2_R as a potential therapeutic for inflammatory respiratory conditions.

The SC, FUB-AMB, is reportedly over 80 times as potent at the CB_1_R as THC (Ivanov et al., 2019) in addition to a 9-13-fold greater affinity for the CB_2_R compared to CB_1_R (Gamage et al., 2018). FUB-AMB was reportedly involved in multiple mass casualties and “zombie outbreaks” from New York to New Zealand (Adams et al., 2017; Gamage et al., 2018; Ivanov et al., 2019). It is also possible that SCs have additional unknown receptor selectivity and binding affinity themselves, or by their metabolites (Trecki et al., 2015; Gamage et al., 2018) with non-cannabinoid receptors (Jinwala and Gupta, 2012) setting the stage for an unpredictable experience. In addition to the unpredictability of the synthetic compound, the vehicle or carrier oil it is in can also have a variety of additional compounds as well. Everything from THC, cannabidiol (CBD), nicotine, caffeine, and tocopherol – a class of compounds containing vitamin E that was associated with multiple hospitalizations from vaping (Dresen et al., 2010) – have been found in mixtures said to contain SCs. These adulterations with additional ingredients lead to misidentification of the substance being used by the consumer and increase the chances for unknown toxicities (Tofighi and Lee, 2012).

## 4. Therapeutic targeting of the endocannabinoid system

Promise with cannabinoids as a therapeutic intervention for respiratory ailments has also been seen as recently as with the COVID-19 pandemic, although with some conflicting outcomes (Pascual Pastor et al., 2020; Malinowska et al., 2021; Paland et al., 2021; Beasley, 2022; Pereira et al., 2022). Recent publications have reported therapeutic cannabis has shown protective effects at preventing contracting COVID-19 (Pascual Pastor et al., 2020; Malinowska et al., 2021; Pereira et al., 2022), the ease of COVID-19 symptom severity (Pascual Pastor et al., 2020), as well as increased susceptibility to COVID-19 infection and exacerbation of COVID-19 symptoms (Paland et al., 2021; Beasley, 2022; Pereira et al., 2022). These contradicting results further highlight the importance and need of further research aimed at understanding all the ways in which cannabis and the EC system can be utilized therapeutically, and where possible cultivation manipulations stand to increase the safety of cannabis consumption through targeted manipulations in the cannabinoid makeup and profiles of cultivated cannabis.

Another potential mechanism for the treatment of a vast array of respiratory ailments comes from the role the central CB_1_R plays in O_2_ saturation and its impact on respiratory rate (Iring et al., 2017). This means pharmacological manipulations to the respiratory system through altered endogenous cannabinoid availability may be plausible treatments for respiratory conditions that involve low levels of O_2_ saturation or irregular breathing patterns. Cannabinoid reuptake inhibitors are becoming another area of promise to increase endogenous cannabinoid concentrations by increasing the available upstream synthesizing enzymes available to produce the endogenous ligands (Hülsmann et al., 2000). While previous clinical trials found adverse effects from brain penetrant CB_1_R antagonists (Nguyen et al., 2019), future drug development may hold the key to well tolerated central CB_1_R antagonists for use by humans (Lazary et al., 2011). Additionally, administration of synthetic cannabinoid agonists and antagonists offer a similar potential outcome for means of treatment of respiratory ailments.

Further research has begun to dive into the pharmacodynamics of cannabis terpenes and their analogs (LaVigne et al., 2021; Liktor-Busa et al., 2021). With over 500 independent compounds identified to exist within cannabis (Rock and Parker, 2021) and more than 700 cultivated varieties (Hazekamp and Fischedick, 2012) that all offer a unique combination of cannabinoid compounds and concentrations. With some of the most common compounds, CBD, THC, and beta-caryophyllene to name a few, showing effects in conditions of pain or anxiety (Gertsch et al., 2008; LaVigne et al., 2021; Soliman et al., 2021), the full scope of outcomes and future therapeutics from specific poly-cannabinoid compositions are only beginning to be investigated. It will be important to continue investigations with these cannabinoids individually and in conjunction with other compounds, as some synergistic actions are found between some cannabinoids and other drugs, such as opioids, that allow for reductions in necessary dosing to achieve pain relief (Fattore et al., 2005; Abrams et al., 2011; Scavone et al., 2013; Bachhuber et al., 2014; Kral et al., 2015; Kazantzis et al., 2016; Lucas and Walsh, 2017; Maher et al., 2017; Hughes et al., 2018; Liang et al., 2018; Wiese and Wilson-Poe, 2018; Lake et al., 2019; Liktor-Busa et al., 2021). Our current understanding of these compounds is promising at possible future potential therapeutic targets able to influence the EC system, and other systems through physiological agonism/antagonism modulation, such as is seen with cannabinoid terpenes that can directly modulate cannabinoid receptor activity through actions on the receptors themselves or through off target influence, as seen with such activity at the TRP channels or on the adenosine system (LaVigne et al., 2021).

Another potential area of promise is the interaction between the EC system and the opioid system. While the receptors of both systems are of the GPCR family and result in inhibition of neuronal activity (Bushlin et al., 2010; Quirion et al., 2020; Zhang et al., 2020), there are some key differences that highlight a potential point of intervention to reduce the negative side effects of opioids, that include the escalating number of fatal overdoses seen with the current overdose epidemic. The most notable difference is the location of receptors, specifically within the pBc, where the location of receptors on the pre- and postsynaptic neuron can completely abolish the burstlet activity of the pBc CPG that reflexively controls breathing (Sun et al., 2019), while CB_1_Rs are only found presynaptically, preventing complete inhibition of this vital respiratory nuclei (Alon and Saint-Fleur, 2017; Iring et al., 2017; Wiese et al., 2021). Furthermore, microglial CB_2_Rs appear to have an ability to override some of this inhibition offering another point of intervention to prevent the fatal effects seen from over ingestion of opioids currently (Wiese et al., 2020).

The hyperpolarization of pBc neurons following MOR activation increases the extracellular K^+^ (Montandon et al., 2016; Varga et al., 2020) and may sufficiently activate nearby microglia, switching from M0 to M1 phenotype. CB_2_R activation can facilitate the anti-inflammatory effects of microglia through downstream cascade events. The anti-inflammatory effects of CB_2_R activation are regulated through microglial polarization (switch from M1 to M2 microglia phenotype), demonstrating a switch from a pro-inflammatory to an anti-inflammatory state (Mecha et al., 2015; Komorowska-Müller and Schmöle, 2020). Use of THC in multiple sclerosis has been shown to increase TNF-α, congruent with an anti-inflammatory state (Bradford and Bradford, 2016). In addition to the established modulatory role that microglia play in the pBc (Hülsmann et al., 2000; Erlichman and Leiter, 2010; Huxtable et al., 2010), it is likely the activation of microglial CB_2_Rs is necessary for respiratory modulation and the physiological antagonism of MOR agonism in the pBc that would otherwise inhibit inspiration. Microglia are activated by opioid administration *via* toll-like4 and toll-like9 receptor agonism (de Oliveira et al., 2022; Reusch et al., 2022). Activation of microglia initiates proinflammatory responses as a result (Merighi et al., 2012). Co-administration of CB_2_R agonists with opioids has shown to reduce opioid induced proinflammatory responses (Tumati et al., 2012) and to be synergistic as pain therapeutics across acute, neuropathic, and complex pain states (Grenald et al., 2017; Yuill et al., 2017). Thus, selective CB_2_R agonism mitigation of opioid-induced respiratory depression by inhibiting microglial activation (Camacho-Hernández et al., 2019) to resynchronize pBc neurons is plausible. Growing evidence suggests that glia-derived proinflammatory mediators enhance tolerance to the anti-nociceptive properties of MOR activation (Watkins et al., 2009). Antagonizing these pro-inflammatory mediators, such as IL-1β, IL-6 and TNF-α, attenuate the development of MOR induced tolerance as well as attenuation of opioid withdrawal induced hyperalgesia (Raghavendra et al., 2002, 2004; Cui et al., 2006) and may be related to the explanation of downstream effects that allow for CB_2_R mitigation of opioid induced respiratory depression. Moreover, endogenous CB_2_R ligands could create a physiological antagonism to opioid induced desynchronization of pBc neurons (Zhang et al., 2017).

Recent publications have shown an opposing role of central versus peripheral CB_1_R activation, with coadministration of the peripherally restricted CB_1_R agonist, PrNMI, and morphine, morphine-induced respiratory depression was completely prevented, while administration of the brain penetrant CB_1_R agonist, AM356, alongside morphine enhanced the already seen respiratory depression (Wiese et al., 2021). Conversely, administration of the brain penetrant CB_2_R agonist, AM2301, in combination with morphine was also able to prevent morphine induced respiratory depression, while the peripherally restricted CB_2_R agonist, AM1710, was not (Wiese et al., 2020), supporting the CB_2_Rs ability to prevent respiratory depression to be completely mediated through central CB_2_Rs. In another study the administration of AM404, an EC reuptake inhibitor, in wild-type and CB_1_R KO mice uncovered the CB_1_R dependent manner of respiratory depression and arterial hypoxia, further supporting limitations of brain penetrant CB_1_R agonists and reuptake and hydrolysis inhibitors (Iring et al., 2017).

## 5. Conclusion

In this review we explored the respiratory system in the context of central versus peripheral control and how the EC system is currently known to influence that control. Next, we reviewed the literature available on organic and synthetic cannabinoids effects on breathing and how that has shaped our understanding of the role the EC system has in respiratory homeostasis. Finally, we looked at some potential future therapeutic applications the EC system has to offer for treatment of respiratory diseases and a possible role in preventing future opioid overdose fatalities that result from respiratory arrest or persistent apnea.

It will be important to fully characterize the cell type and location within the central respiratory nuclei, as well as in the periphery if viable therapeutics are going to be developed. It will also be vital to understand dose response curves and off target binding affinity for other cannabinoid receptors, or even more selective agonists. Specifically in the case of the CB_1_R, as it appears to have opposed roles in the periphery and central nervous system. This means understanding the dosing of peripherally restricted CB_1_R agonists to ensure they do not cross the blood brain barrier will also be of high importance for public safety since central CB_1_R activation enhances respiratory depression instead of mitigating it when administered alongside opioids (Wiese et al., 2021). Additionally, with the similarities in the cannabinoid receptors, many ligands will spill over to bind the other cannabinoid receptor once the intended targets are all full or activate the other cannabinoid receptor in conditions of genetic deletions of the intended cannabinoid receptor (Wiese et al., 2020). This is what was seen with escalating doses of CB_2_R agonists administered alongside morphine. The CB_2_R agonist began to leak over and activate central CB_1_Rs at the same time, causing enhanced respiratory depression and abolishing the protective feature of central CB_2_R activation alongside morphine. The ability to mitigate morphine induced respiratory depression through CB_2_R activation appears to be mediated centrally, as these effects have not been shown through activation of peripherally restricted CB_2_R. Activation of CB_2_R plays an additional role in modulating the immune system through the release of anti-inflammatory factors. There may also be a role for immunosuppression as studies across several disease states have shown downstream effects of cannabinoid receptor activation to include induction of apoptosis, inhibition of cell proliferation, inhibition of cyto- and chemokine production, reduced cytokine activation, T cell proliferation, and induction of regulatory T lymphocytes. With the expansion of cannabis access, it is essential that investigations continue to uncover the underpinnings and mechanistic workings of the EC system, the impact cannabis, and exogenous cannabinoids have on these systems, and how some of these compounds can mitigate respiratory depression when combined with opioids.

Respiratory control is complex and begins in the brainstem without peripheral input (Del Negro et al., 2018). The key regions are coordinated through a CPG, the pBc, a component of the ventral respiratory group that interacts with the dorsal respiratory group to synchronize burstlet activity and produce inspirations (Ghali, 2019). An additional rhythm generator: the retrotrapezoid nucleus (Eljaschewitsch et al., 2006)/parafacial respiratory group drives active expiration during conditions of exercise or high CO_2_ (Janczewski and Feldman, 2006). Combined with the feedback information from the periphery: through carotid bodies, stretch of the diaphragm or intercostal muscles, chemo- and baroreceptors, lung tissue, immune cells, and the cranial nerves, our respiratory system can fine tune motor outputs that ensure we have the O_2_ necessary to survive and can expel the CO_2_ waste we produce. It is important that we understand all the ways we can treat and protect our respiratory system to ensure its ability to function for the duration of our lifetime. It is vital to public safety that we understand the effects these compounds have on lung tissue and function, as well as how our endogenous cannabinoids influence our respiratory behavior for future therapeutic discovery.

## Author contributions

BW and AAR contributed equally to the writing and revisions and comments were provided by TV and TL-M funding. All authors contributed to the article and approved the submitted version.

## Funding

This work was funded by R01DA056608 to TL-M and TV, Comprehensive Pain and Addiction Center, and Department of Pharmacology NIH/NIDA 1P30DA051355.

## Conflict of interest

The authors declare that the research was conducted in the absence of any commercial or financial relationships that could be construed as a potential conflict of interest.

## Publisher’s note

All claims expressed in this article are solely those of the authors and do not necessarily represent those of their affiliated organizations, or those of the publisher, the editors and the reviewers. Any product that may be evaluated in this article, or claim that may be made by its manufacturer, is not guaranteed or endorsed by the publisher.

## Figures and Tables

**Table 1:** 

pBc	preBötzinger complex
CB_1_R	cannabinoid receptor 1
CB_2_R	cannabinoid receptor 2
SC	synthetic cannabinoid
EC	endocannabinoid
CNS	central nervous system
CPG	central pattern generator
NK1	neurokinin-1 receptor
MOR	mu opioid receptor
NMDA	*N*-methyl-D-aspartate
DBX1	developing brain homeobox 1 receptor
PPARS	peroxisome proliferator-activated receptors
K^+^	potassium
MAPK	mitogen-activated protein kinase
AEA	anandamide
2-AG	2-arachidonoylglycerol
KO	knockout
ERK1/2	extracellular signal-regulated kinases ½
O_2_	oxygen
CO_2_	carbon dioxide
P_O2_	partial oxygen
P_CO2_	partial carbon dioxide
THC	tetrahydrocannabinol
CBD	cannabidiol
